# Saving Moore’s Law Down To 1 nm Channels With Anisotropic Effective Mass

**DOI:** 10.1038/srep31501

**Published:** 2016-08-19

**Authors:** Hesameddin Ilatikhameneh, Tarek Ameen, Bozidar Novakovic, Yaohua Tan, Gerhard Klimeck, Rajib Rahman

**Affiliations:** 1 Department of Electrical and Computer Engineering, Purdue University, USA

## Abstract

Scaling transistors’ dimensions has been the thrust for the semiconductor industry in the last four decades. However, scaling channel lengths beyond 10 nm has become exceptionally challenging due to the direct tunneling between source and drain which degrades gate control, switching functionality, and worsens power dissipation. Fortunately, the emergence of novel classes of materials with exotic properties in recent times has opened up new avenues in device design. Here, we show that by using channel materials with an anisotropic effective mass, the channel can be scaled down to 1 nm and still provide an excellent switching performance in phosphorene nanoribbon MOSFETs. To solve power consumption challenge besides dimension scaling in conventional transistors, a novel tunnel transistor is proposed which takes advantage of anisotropic mass in both ON- and OFF-state of the operation. Full-band atomistic quantum transport simulations of phosphorene nanoribbon MOSFETs and TFETs based on the new design have been performed as a proof.

Shrinking the size of metal oxide semiconductor field effect transistors (MOSFETs) has improved the functionality, speed, and cost of microprocessors over the last four decades. However, the advantages of scaling are quickly fading away^1^. For example, the operational frequency of CPUs has stopped improving since 2003 due to power consumption of CPUs reaching their cooling limit (≈100 W/cm^2^)^2^. Moreover, scaling down *L*_*ch*_ towards the few nanometer regime is becoming more challenging due to source-to-drain (SD) leakage current^3^,^4^; the gate controlled potential barrier becomes more transparent as channel becomes shorter and direct SD tunneling increases. Another challenge in miniaturizing MOSFETs is scaling down the supply voltage *V*_*DD*_^2^. A smaller *V*_*DD*_ can be achieved in a switch with sharper ON to OFF transition. However, the steepness of conventional MOSFETs have a fundamental limit due to thermionic injection of carriers over the channel barrier (60 *mV*/*decade* at room temperature). Accordingly, *V*_*DD*_ in MOSFETs does not scale very well. On the other hand, tunnel FETs (TFETs) can, in principle, provide steeper switching^5^,^6^. Nevertheless, scaling TFETs is even trickier than MOSFETs, since scaling affects both ON- and OFF-states of the TFETs^7^,^8^,^9^. Hence, the tremendous improvement in processing power of transistors every few years linked to the dimension scaling and empirically described by Moore’s law has reached a dead end. Fortunately, it is shown here that 2D materials with anisotropic effective mass (m*) can be used to solve these problems and save Moore’s law.

First, we discuss the source-to-drain tunneling challenge of the ultra scaled MOSFETs. Reducing the channel size makes the potential barrier more transparent. To visualize this, the transmission is shown in colormap on a logarithm scale and with an overlayed band diagram of MOSFETs in Fig. 1. The band diagram and transmission profile of a 12 nm and 5 nm long channel GaAs MOSFET are compared respectively in Fig. 1a,b. 5 nm long channel GaAs MOSFET suffers significantly from SD leakage which reduces the gate control. Equation (1) shows the dependence of tunneling current through barrier on m* of the channel material^4^,^10^,^11^,^12^,^13^. According to Equ. (1), an apparent solution to the high transparency of channel barriers in short channel regime is a channel material with higher effective mass.


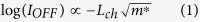


Although high m* channel materials block SD tunneling effectively, they have a set of drawbacks too. Quantum capacitance (*C*_*Q*_) of channel material increases as a result of larger density of states (DOS) and m*. Accordingly, the gate capacitance (*C*_*G*_) which is the net series capacitance of *C*_*Q*_ and oxide capacitance (*C*_*ox*_) increases. Hence, a larger m* translates into a larger switching delay (*τ* = *C*_*G*_*V*_*DD*_/*I*_*ON*_).


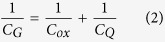


Anisotropic effective mass can provide a solution to this problem with reducing *C*_*Q*_ by a factor of 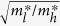. This reduction of *C*_*Q*_ is the result of the decreased density of states (*DOS*) in anisotropic materials:


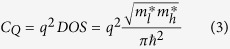


where 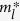 and 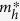 are low and high effective masses of the channel material along its two main axes. If high m* axis of channel is aligned with transport direction and low m* axis is aligned with the confinement direction, both low transparency and small switching delay can be achieved. Note that high m* along the channel increases the carriers decay rate through barrier exponentially, whereas low confinement m* reduces DOS and *C*_*Q*_. Hence, a 2D material such as phosphorene^14^ with anisotropic m*^15^ can provide an excellent switching performance in MOSFETs ensuring the continuation of Moore’s Law to atomic dimensions.

Here, we discuss the scaling challenge of TFETs. Although TFETs were intended to reduce the power consumption of transistors^5^,^6^, scaling TFETs below 10 nm is even more challenging than MOSFETs^7^,^8^,^9^. The ON-state and OFF-state tunneling currents (I_ON_ and I_OFF_) depend on the same device parameters^12^. Thus decreasing I_OFF_ would reduce I_ON_. Roughly, the ON/OFF ratio of TFETs depends on^8^,^12^,^13^,^36^:


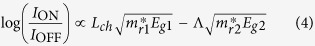


where Λ and *L*_*ch*_ are the tunneling distances in the ON- and OFF-state respectively. 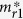 and *E*_*g*1_ (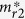 and *E*_*g*2_) are the reduced effective mass and the bandgap of the channel material (source-to-channel junction), respectively.

Shrinking the channel length to few nanometers brings *L*_*ch*_ close to Λ and reduces I_ON_/I_OFF_ significantly. One apparent solution can be a heterostructure channel where the term 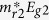 is much smaller than 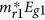 due to different materials used in the source and channel regions^16^,^17^. However, heterostructure TFETs suffer from interface states which deteriorate their OFF-state performance^18^,^19^,^20^. Although homojunction TFETs do not have the interface states, it is challenging to provide high ON/OFF ratio especially below 6 nm^8^,^21^. Anisotropic effective mass can also provide a solution for this challenge by setting source-channel junction along low m* axis of channel material and the channel barrier along high m* axis. In this work, a novel TFET is proposed which works based on homojunction channel and anisotropic m* and hence it is free of interface states between different channel materials in heterojunctions.

Although, many novel materials and designs have been proposed to enhance the performance of TFETs such as 2D material TFETs^22^,^23^,^24^, Nitride heterostructures^16^, dielectric engineering^25^, there are not many proposals for solving the scaling challenge of TFETs^8^. In this work, a new TFET design is proposed to overcome the scaling challenge and enable downsizing to 2 nm channel lengths. Figure 2a shows a novel TFET device structure to take advantage of anisotropic effective mass. Notice that the gate is L-shaped. Figure 2b depicts that the tunneling in the ON-state occurs along the low m* axis of the channel enhancing the I_ON_. However, the tunneling in the OFF-state occurs along the high m* axis and results in a very low I_OFF_. Hence, this new TFET design can revive Moore’s law for sub-10 nm TFETs.

In this work, phosphorene nanoribbon has been chosen as the channel material since it has a large effective mass anisotropy in zigzag and armchair directions. The electron and hole effective mass values of phosphorene along zigzag and armchair directions calculated from our tight-binding method are compared with the DFT calculations from literature^26^,^27^ in Table 1. Moreover, multi-layer phosphorene provides a range of bandgap (*E*_*g*_ ≈ 1.45 to 0.4 eV^28^,^29^) suitable for transistor applications. *E*_*g*_ of monolayer (1L-) and bilayer (2L-) phosphorene is about 1.45 eV and 0.8 eV, respectively. Since MOSFETs require larger *E*_*g*_ for a smaller source-to-drain leakage, a monolayer phosphorene has been used here. The situation is more tricky in TFETs which need optimized *E*_*g*_. It was shown previously that 2L-phosphorene has an optimum *E*_*g*_^21^, and hence 2L has been chosen for TFETs. HfO_2_ is used as the gate dielectric with an equivalent oxide thickness (EOT) of 0.5 nm in both MOSFETs and TFETs and I_OFF_ is set to 10^−4^ *μA*/*μm*.

Figure 3a compares *I*_*DS*_-*V*_*GS*_ of a conventional 2L-phosphorene nanoribbon along zigzag and armchair transport directions with L-shaped gate (L-gate) TFET calculated from full-band atomistic quantum transport simulations using NEMO5^30^,^31^. Not only does the L-gate TFET have I_ON_ close to that of the armchair ribbon (low m*), but it also has I_OFF_ similar to that of the zigzag ribbon (high m*). Hence, the L-gate design has the advantages of both low and high m* devices simultaneously: high I_ON_ and low I_OFF_.

The performance of the L-gate TFET depends on the length *dL* (see Fig. 3b) which determines the width for ON-current. In conventional TFETs, *dL* equals 0. Figure 3b shows I_ON_ of L-gate TFET as a function of *dL* for a fixed I_OFF_ of 10^−4^ *μA*/*μm*. Increasing *dL* enhances I_ON_ significantly. The larger I_ON_/I_OFF_ ratio translates into a lower SS as shown in Fig. 3b. However, increasing *dL* reduces the source extension by *dL*/2. Accordingly, there is a limit on *dL* according to the footage requirements in the design. Nevertheless, a *dL* of about 2.5 nm can improve the performance of TFET approximately by 2 orders of magnitude.

Figure 3c shows ultra-scaled L-gate TFETs with channel lengths from 9 nm down to 2.3 nm with a *V*_*DD*_ of 0.2 V. In ultra-scaled TFETs, *V*_*DD*_ cannot scale below *V*_*DD*_ = 0.2 *V* since the maximum tunneling energy window is limited by *V*_*DD*_. The L-gate TFETs with *L*_*ch*_ above 2 nm provide I_ON_/I_OFF_ > 10^4^ and satisfy the minimal ITRS requirement for I_ON_/I_OFF_ ratio. Although the L-gate design has improved the performance of TFETs significantly, the ON/OFF ratio of TFET decreases for devices with *L*_*ch*_ and *V*_*DD*_ below 2 nm and 0.2 V, respectively.

Figure 3d shows I_ON_/I_OFF_ ratio of L-gate TFETs as a function of *L*_*ch*_. Ultra-scaled channel lengths put a limit on *dL*. Hence, *dL* shrinks down from 3.5 nm to 1 nm when the channel length scales down from 9 nm to 2.3 nm. L-gate TFETs with channel lengths down to 2 nm provide I_ON_/I_OFF_ ratio larger than 10^4^ (required by ITRS as minimum amount of I_ON_/I_OFF_ ratio). This result proves that L-gate TFETs with a channel material of anisotropic m* enable successful scaling of TFETs down to the ultimate limit; a channel with a few atoms.

The unpaired bonds at the edges of phosphorene nanoribbon can introduce metallic edge states^32^,^33^. To avoid these edge states, all the unpaired phosphorene bonds have to be properly passivated (e.g. by hydrogen). In this work, a hydrogen passivation model for tight-binding basis is used to passivate the dangling bonds^34^. It is also possible to create an edge-less L-gate structure as shown in Fig. 3e. This structure pattern is created by repeating and mirroring the original L-gate pattern providing the same current density per unit width compared with the original design.

As mentioned before, ultra-scaled MOSFETs require large m* and *E*_*g*_ to block source-to-drain tunneling. Hence, 1L-phosphorene nanoribbon has been chosen here which has the highest m* and *E*_*g*_ compared to multilayer phosphorene. The schematic of the 1L-phosphorene MOSFET has been shown in Fig. 4a. The supply voltage is fixed to 0.5 V, much higher than *V*_*DD*_ of TFETs, since the Boltzmann limit of subthreshold swing in MOSFETs (i.e. 60 mV/decade in room temperature) does not allow the scaling of *V*_*DD*_.

The transfer characteristics of a short channel 1L-phosphorene (*L*_*ch*_ = 3 nm) with transport direction along low m* (armchair) and high m* (zigzag) are compared in Fig. 4b. As expected, the gate efficiency of a phosphorene MOSFET is much better when the high m* (zigzag) axis is along the transport direction. This better gate efficiency improves the subthreshold slope of MOSFET significantly.

Figure 4c shows *I*_*DS*_-*V*_*GS*_ of zigzag scaled phosphorene MOSFETs with channel lengths from 12 nm to 1.6 nm. Notice that for phosphorene MOSFETs with *L*_*ch*_ > 1.6 *nm* an *I*_*ON*_ larger than 1.1 *mA*/*μm* and an I_ON_/I_OFF_ ratio larger than 10^6^ have been achieved. 1L-phosphorene MOSFETs show a significant advantage over other 2D materials whose performances are diminished below 5 nm channel lengths^35^.

MOSFETs with long channels do not suffer from source-to-drain tunneling. Accordingly, a high transport m* is not required for blocking this leakage current. Actually, in long channel regime, a low transport m* can be beneficial and enhance the ON-state performance of the transistor since it leads to a higher carrier injection velocity. Figure 4d shows I_ON_/I_OFF_ ratio of phosphorene nanoribbon MOSFETs as a function of *L*_*ch*_ along zigzag and armchair transport directions. Although zigzag nanoribbon MOSFETs significantly outperform the armchair ones in short channels due to lower source-to-drain tunneling, armchair nanoribbon MOSFETs show a better performance in longer channels due to higher injection velocity. There is a critical channel length (i.e. 6 nm in 1L-phosphorene) in MOSFETs below which having a high m* becomes critical and above which a low m* is beneficial.

In summary, the channel materials with anisotropic effective mass can be used to design transistors scalable to 1–2 nm channel lengths. In MOSFETs, the high effective mass along transport direction blocks the direct source to drain tunneling and low effective mass reduces the quantum capacitance and switching delay. On the other hand in TFETs, a novel L-shaped gate design is proposed which can provide advantage of high tunneling rate in the ON-state and low tunneling rate in OFF-state by engineering the tunneling paths along low and high effective mass directions. In summary, anisotropic effective mass can be used in an L-gate design to obtain large ON/OFF ratio in an ultra-scaled homojunction TFET.

## Methods

The atomistic quantum transport simulation results have been obtained from the self consistent solution of 3D-Poisson equation and Non-equilibrium Green’s Functions (NEGF) method using the Nanoelectronics modeling tool NEMO5^30^,^31^. The Poisson equation provides the potential for NEGF method and takes the free charge in return. The tight-binding Hamiltonian of phosphorene used in NEGF calculations employs a 10 bands *sp*^3^*d*^5^s* model. Phosphorene is a material with anisotropic dielectric properties. The details of the Poisson equation with anisotropic dielectric tensor and NEGF equations can be found in our previous works^21^,^22^,^36^,^37^.

## Additional Information

**How to cite this article**: Ilatikhameneh, H. *et al*. Saving Moore’s Law Down To 1 nm Channels With Anisotropic Effective Mass. *Sci. Rep.* **6**, 31501; doi: 10.1038/srep31501 (2016).

## Figures and Tables

**Figure 1:**
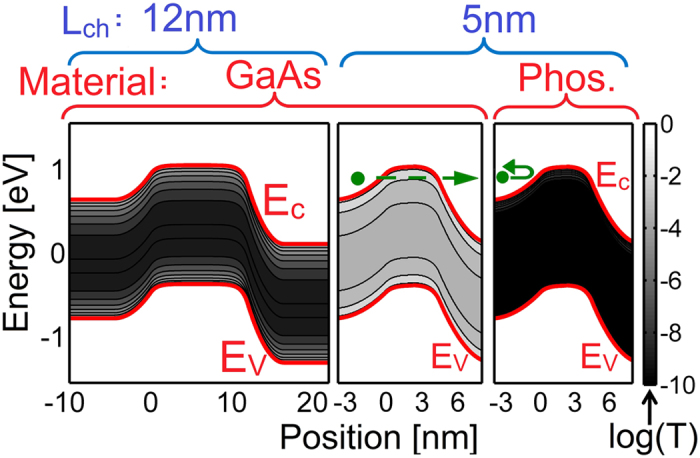
The band diagram of (**a**) 12 nm long GaAs, (**b**) 5 nm long GaAs, and (**c**) 5 nm long phosphorene MOSFETs. The colormap shows the transparency of the channel. The potential barrier in the 5 nm long GaAs MOSFET is transparent and hence, the gate efficiency is low. This problem can be solved by using phosphorene with high m*.

**Figure 2:**
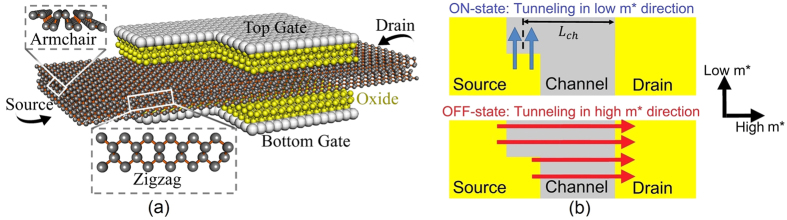
(**a**) The device structure of the bilayer phosphorene TFET with L-shaped gate. (**b**) The main tunneling paths in the ON-state (blue arrows) and OFF-state (red arrows) of the phosphorene TFET.

**Figure 3:**
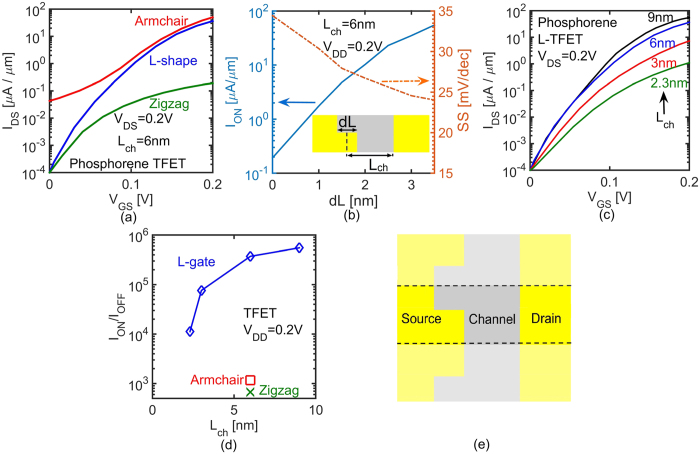
(**a**) The comparison between *I*_*DS*_-*V*_*GS*_ of conventional 2L-phosphorene nanoribbons along zigzag and armchair directions with that of the L-gate TFET. (**b**) ON-current and SS of L-gate TFET as a function of *dL*. (**c**) Impact of channel length scaling on *I*_*DS*_-*V*_*GS*_ of L-gate TFETs. (**d**) I_ON_/I_OFF_ ratio of the L-gate TFET as a function of *L*_*ch*_. (**e**) The top view of edge-less L-gate design.

**Figure 4:**
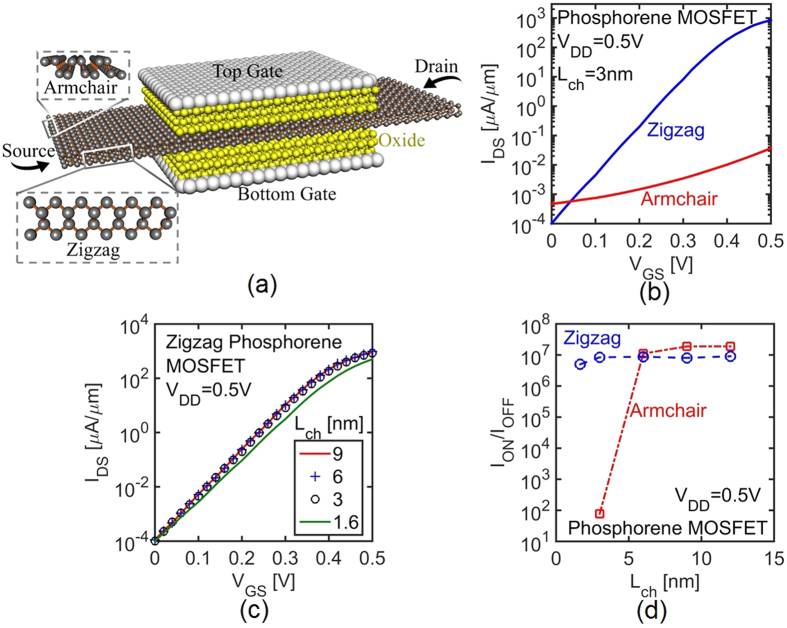
(**a**) Device structure of zigzag monolayer phosphorene MOSFET. (**b**) The comparison between *I*_*DS*_-*V*_*GS*_ of phosphorene nanoribbon MOSFETs with transport direction along high m* (zigzag: blue) and low m* (armchair: red) axes. (**c**) Impact of *L*_*ch*_ scaling on *I*_*DS*_-*V*_*GS*_ of phosphorene MOSFETs. (**d**) I_ON_/I_OFF_ ratio of MOSFETs as a function of *L*_*ch*_ along zigzag and armchair transport directions.

**Table 1:** The electron and hole effective mass values of phosphorene along armchair and zigzag directions calculated from our tight-binding method (TB) and DFT calculations from literature (HSE06^26^ and PBE^27^).

	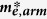	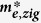	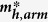	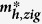	Ref.
1L	0.17	1.09	0.15	5.84	TB
	0.17	1.12	0.15	6.35	HSE06^26^
	0.14	1.23	0.13	13.09	PBE^27^
2L	0.17	1.13	0.14	2.8	TB
	0.18	1.13	0.15	1.81	HSE06^26^
	0.11	1.35	0.1	2.18	PBE^27^
